# Whole body diffusion for metastatic disease assessment in neuroendocrine carcinomas: comparison with OctreoScan® in two cases

**DOI:** 10.1186/1477-7819-10-82

**Published:** 2012-05-16

**Authors:** Rachel Jorge D Cossetti, Regis Otaviano França Bezerra, Brenda Gumz, Adriana Telles, Frederico P Costa

**Affiliations:** 1 Centro de Oncologia, Hospital Sírio Libanês, Rua Dona Adma Jafet 91, São Paulo, 01308-050, Brazil

**Keywords:** Neuroendocrine tumor, Diffusion-weighted image, Magnetic resonance image, OctreoScan®

## Abstract

Neuroendocrine tumor (NET) patients must be adequately staged in order to improve a multidisciplinary approach and optimal management for metastatic disease. Currently available imaging studies include somatostatin receptor scintigraphy, like OctreoScan®, computed tomography (CT), scans and magnetic resonance imaging (MRI), which analyze vascular concentration and intravenous contrast enhancement for anatomic tumor localization. However, these techniques require high degree of expertise for interpretation and are limited by their availability, cost, reproducibility, and follow-up imaging comparisons. NETs significantly reduce water diffusion as compared to normal tissue. Diffusion-weighted imaging (DWI) in MRI has an advantageous contrast difference: the tumor is represented with high signal over a black normal surrounding background. The whole-body diffusion (WBD) technique has been suggested to be a useful test for detecting metastasis from various anatomic sites. In this article we report the use of DWI in MRI and WBD in two cases of metastatic pulmonary NET staging in comparison with OctreoScan® in order to illustrate the potential advantage of DWI and WBD in staging NETs.

## Background

Neuroendocrine tumors (NETs) are heterogeneous malignant neoplasms that originate from neuroendocrine cells located in various anatomic sites of the body. They are classically associated with symptoms resulting from secretion of hormones or vasoactive peptides into the systemic circulation, and classified as functioning and non-functioning tumors. The Surveillance, Epidemiology, and End Results Program database reported the incidence of 5.25 cases per 100,000 for the 2004 United States population [1]. The long survival experienced by NET patients explains its high prevalence, which was estimated in 35/100,000 in the same population. Approximately 50 % of NET patients are diagnosed with localized disease and surgical resection alone is often curative.

The liver is a frequent site of metastasis, with substantial influence on prognosis [2-5]. Patients who present with advanced disease limited to the liver may also benefit from surgical resection with potential curative intent or to control hormone secretion refractory to biotherapy or other systemic treatment options. In order to better select candidates for surgical procedure, clinical staging is fundamental.

Despite great technological improvement in imaging acquisition in recent years, detection of hepatic lesions less than 5 mm in diameter is still limited. Therefore, diffusion-weighted imaging (DWI) in MRI has recently emerged as a tool for detecting metastasis in the liver without the need of i.v. contrast or radiation exposure [6,7]. DWI sequence can be adjusted to obtain images from the entire body in one single acquisition called whole-body diffusion imaging (WBD). To our knowledge, only limited studies have addressed the value and the practicality of MRI diffusion in staging NETs [8,9]. The objective of this report is to assess the DWI and WBD in two patients with metastatic neuroendocrine carcinoma and demonstrate its potential use in comparison with OctreoScan®, commonly used in the clinical staging of NET.

### Case presentation

We report two patients with metastaic neuroendocrine carcinoma submitted to MRI with DWI and WBD and OctreoScan® in their clinical staging. The imaging protocols and medical history is presented bellow.

### MRI image protocol

MRI was performed with a 1.5-T whole-body imager (Signa HDx; General Electric Medical Systems, Milwaukee, WI, USA). For every scan, whole-body examination was obtained in axial and coronal plane using body coil. Three sequences were obtained and the image parameters are described in the Table 1.

**Table 1:** Whole-body magnetic scan parameters: all images were obtained using a body coil and with free-breathing technique

**Parameter**	**DWI**	**T1**	**STIR**
TR/TE	6750/64	1000/4.6	3200/60
Inversion time (ms)	180	-	180
Field of view (mm)	450	450	450
Matrix size	100 × 128	256 × 224	256 × 128
Section thickness/gap (mm)	5	5	5
Parallel acquisition technique factor	-	-	-
Signals averaged (*n*)	6	1	2
Blocks (*n*)	5	5	5
Acquisition time	2 min 30 s for each block	32 s for each block	1 min 40s for each block
B-value (s/mm2)	0, 600	-	-
Bandwidth (kHz)	125	62.5	50

Dash (-) indicates not applicable.

DWI, diffusion-weighted imaging; STIR, Short T1 Inversion Recovery.

For each sequence, at least five continuous stations covering the whole body from the top of the head to feet were acquired. Estimated examination time is up to 30 min.

The diffusion encoding was done in only one direction and the lesions detected in the diffusion-weighted sequence were analyzed in terms of number, size, location, and signal intensity. These lesions were compared to the T1-weighted and STIR (Short TI Inversion Recovery) sequences to rule out benign lesions (false-positive).

Using quantitative analysis, an apparent diffusion coefficient map (ADC map) was calculated for the main lesions in each patient. After these analyses, all lesions considered malignant were correlated with OctreoScan® with time interval between the exams of less than one week.

### Somatostatin receptor scintigraphy

Somatostatin receptor scintigraphy (SRS) is a functional technique for the imaging of NETs. There is a variety of peptides (known as somatostatin analogues) which target these receptors, and which differ in their ability to bind to the various receptor subtypes (SSTR). The most commonly used somatostatin analogue is octreotide which is labeled with 111In, using the chelator diethylenetriamine-pentaaceticacid (DTPA), to produce 111In-DTPA-octreotide [6-11] which is available as a commercial product (OctreoScan®-Mallinckrodt, Inc.). OctreoScan® was supplied as two vials: Vial A - 111In as InCl3, 122 MBq (3.3 mCi)/1.1 ml; Vial B - 10 μg of lyophilized pentetreotide and excipients. The maximum amount of IV pentetreotide injected is 10 μg.

OctreoScan® images are obtained by using a gamma camera fitted with a medium-energy, parallel hole collimator (Symbia T2, Siemens) with hybrid technique SPECT/CT (single-photon emission computed tomography and digital computed tomography at the same study). Planar and SPECT images were acquired at 4 and 24 h post-injection.

### Medical history

#### Patient 1

**Figure 1:**
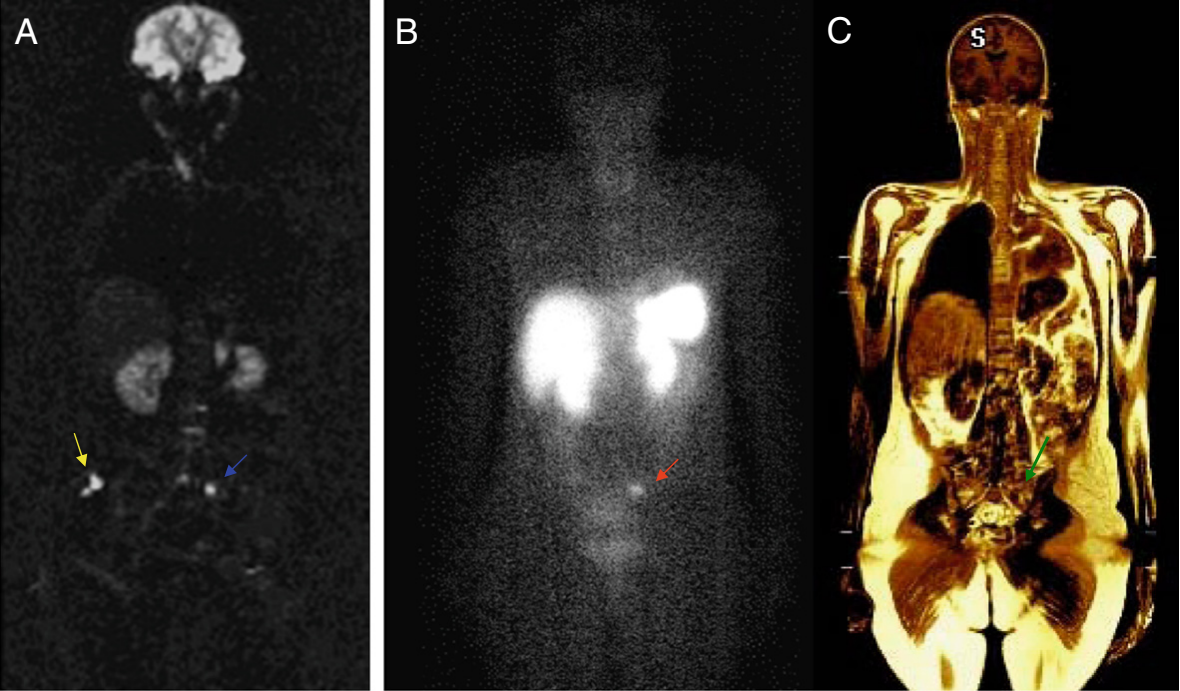
**Comparison of DWI (A), 111 In-pentetreotide scintigraphy images (B) and T1-weighted (C) showing bone metastasis in left sacral bone detected by diffusion-weighted imaging (blue arrow) and confirmed by T1 sequence (green arrow).** The hyperintense signal in the right subcutaneous fluid (yellow arrow) is a pitfall due to T2 shine-throught effect and should not be interpreted as metastasis. OctreoScan® image (**B**) reveals correlation between radiotracer uptake (red arrow) and MR findings.

A 31-year-old female underwent dermatological evaluation for resection of a subcutaneous nodule. Pathological analysis demonstrated an adenocarcinoma with positive margins. The patient underwent wide excision of surgical margins and pathology revealed a NET with positive staining for chromogranin A and neuron-specific enolase. A chest CT scan showed a pulmonary mass in the left lobar bronchia. The patient underwent left pneumectomy. Pathology confirmed a 4.5-cm well-differentiated neuroendocrine carcinoma, with a low mitotic count of 1 mitosis per 10 HPF (high power field). There was no vascular or pleural invasion and surgical margins were free. One lymph node was involved. A staging abdominal ultrasound revealed the presence of two hepatic nodules in segment VIII, measuring 1.8 and 1.0 cm. An abdominal MRI showed multiple hepatic nodules, with additional lesions in segments II and V. Octreoscan showed increased activity on pulmonary and hepatic tumoral lesions. Laboratory screening was unremarkable. The patient was treated with somatostatin analogs. Follow-up abdominal MRI revealed multiple new hepatic and bone lesions. In the WBD images, a new small sacral lesion was detected in correlation with a low signal nodule on the T1-weighted images and therefore raised suspicion for metastasis (Figure 1A and 1 C). The Octreoscan® performed in the same week of the WBD/MRI showed focal uptake in this area (Figure 1B) confirming that WBI added valuable information for staging. Everolimus was added to the treatment regimen. Follow-up images revealed stable disease after three months.

#### Patient 2

**Figure 2:**
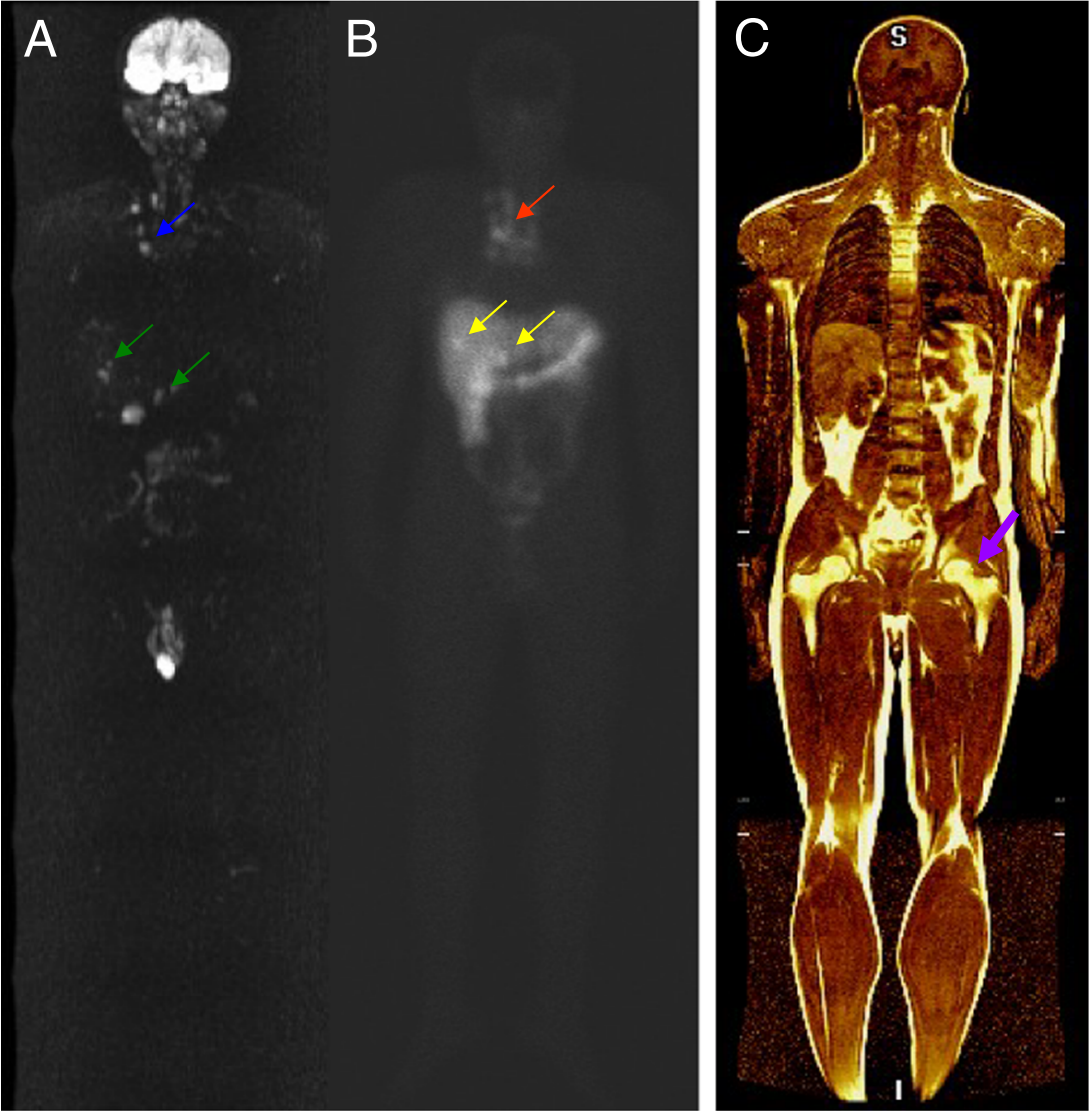
**Comparison of DWI (A) and 111 In-pentetreotide scintigraphy (B) images reveal correlation between hyperintense mediastinal lymph nodes (blue arrows) and radiotracer uptake (red arrow).** All the metastatic liver lesions detected by scintigraphy (yellow arrows) appeared as hyperintense nodules in the DWI (green arrows). T1-weighted image (**C**) shows the potential application of this method to detect bone metastasis (purple arrows).

A 23-year-old male presented with sporadic haemoptysis over the last year. A CT scan of the thorax showed an intra-bronchial lesion with 3.0 × 2.3 cm in the right inferior lobar bronchia and a bronchoscopy confirmed a well-differentiated NET, grade 1. The tumor was a non-functioning pulmonary NET-1. He underwent a right inferior lobectomy. Pathology reported an invasive well-differentiated pulmonary neuroendocrine tumor, grade 1, measuring 2.2 cm, with predominant endobronchial growth, with less than 1 mitosis per 10 HPF. There was neither neural-vascular invasion nor necrosis. According to pathological analysis, zero of two mediastinal lymph nodes were affected (pT1b pN0). Immunohistochemistry revealed a proliferation index (Ki67) of < 1 %, with positive staining for chromogranin A, synaptophysin, cytokeratin(CK)-40, CK-48, CK-50,6 and kDa, and negative staining for TTF-1. A staging CT scan of thorax, abdomen, and pelvis demonstrated unexpected small round hypointense lesions distributed throughout the liver parenchyma, seen only on the venous phase, measuring less than 10 mm. Laboratory exams were relevant for serum chromogranin A 38 ng/mL (normal reference value: < 15 ng/mL) and urinary 5-hydroxyindoleacetic acid (5-HIAA) 5.7 mg/24 h (normal reference value: < 8.2 mg/24 h). The patient was referred for hepatic resection at our center. An abdominal MRI was performed to evaluate the hepatic lesions, revealing multiple hypervascular nodules scattered throughout the liver with washout on the delayed phase. All these hepatic lesions were detected in the DW images. WBD images revealed, in addition to the hepatic lesions, mediastinal lymph nodes with restricted diffusion confirmed in the ADC maps raising suspicion for metastatic lesions (Figure 2A). The Octreoscan® performed on the day after showed focal areas of uptake in superior mediastinal, para-tracheal, subcarinal and right pulmonar hilar lymph nodes and hepatic nodules in segments V/VIII and III (Figure 2B). The patient was submitted to a fine needle aspiration biopsy of the large hepatic lesion, which confirmed metastatic neuroendocrine carcinoma. Treatment with somatostatin analogs was initated.

## Discussion

Magnetic resonance or computed tomography (CT) explore NET characteristics of being highly vascular and enhance intensely with intravenous contrast during the early arterial phases with washout in the delayed phases. On the other hand, liver metastasis may appear isodense in pre-contrast and venous phases causing poor visualization. This variation creates challenges in staging and evaluating tumor response or progression during treatment. Multiphase CT, which includes both arterial and portal venous phase images, is often used to stage and access tumor response in patients with NET. Magnetic resonance imaging (MRI) is also a commonly used alternative for imaging NET. Metastatic lesions can be visualized with and without gadolinium contrast, especially in T2-weighted sequences, reducing the variability sometimes seen with CT-based imaging results [10,11]. However, both imaging methods require intravenous (i.v.) contrast.

Scintigraphic technique is also commonly used to stage NET by identifying primary and metastatic anatomic sites, especially in well and moderately-differentiated NETs. OctreoScan® specifically binds to SSTR, with particular affinity for subtypes 2, 3, and 5, when they are expressed in tumor. ^68^ Ga-DOTANOC is another promising peptide with broader and higher affinity with SSTR. It seems to provide better visualization and relevant information in the management of NET patients compared to OctreoScan®, although this new peptide is still not widely available outside a small number of referring centers in the world [12-14].

NETs significantly reduce water diffusion compared to normal tissues. DWI in MRI has an advantageous contrast difference for lesion detection: the tumor is represented with high signal over a black normal surrounding tissue. This is a patient-friendly tool that does not require i.v. contrast, does not use ionizing radiation, and can be correlated with other T1/T2 MRI acquisitions for better tumor location and characterization. The WBD image is a fast acquisition phase and can be easily used as a roadmap to identify small and large NET lesions. Repeated images can also be made in order to assess tumor response, not only with morphological analysis but also providing functional information through quantification of apparent diffusion coefficient (ADC) values. The precise anatomic information, the speed of imaging acquisition, the lack of endovenous contrast, wide availability, and low cost make the whole body diffusion a reliable and attractive staging method that should be further investigated.

WBD has been shown to be the most accurate technique for detecting metastasis of melanoma in the liver, bone, subcutaneous tissue, and intra-peritoneum [15]. WBD has also been used to detect metastatic lesions from paraganglioma and pheochromocytoma, with higher rates of detection than 2-[(18)F]-fluoro-2-deoxy-D-glucose positron emission tomography (FDG-PET) or (123)I-meta-iodo-benzyl guanidine scintigraphy (MIBG) [16].

Despite the paucity of diffusion MRI reports in NETs, these two cases illustrate a potential role of DWI and WBD as a cost-effective and less invasive method for assessing NETs. Variations in water diffusion restriction could provide information about tumor response during treatment, with less cost and risks in comparison with other standard methods. We acknowledge that the functional information provided by WBD still needs further evaluation and currently does not substitute OctreoScan®. A formal evaluation in NET patients should be conduct to define the sensitivity and specificity of this method in comparison with other standard imaging techniques. DWI and WBD could have a potential and import role for staging NET in surgical candidates and for following up patients on systemic therapy.

### Consent

Written informed consent was obtained from the patients for publication of this case report and any accompanying images. A copy of the written consent is available for review by the Editor-in-Chief of this journal.

## Abbreviations

ADC map, Apparent diffusion coefficient map; CK, Cytokeratin; CT, Computed tomography; DTPA, Diethylenetriamine-pentaaceticacid; DWI, Diffusion-weighted image; FDG-PET, 2-[(18)F]-fluoro-2-deoxy-D-glucose positron emission tomography; HPF, High power field; i.v., Intravenous; MIBG, (123)I-meta-iodo-benzyl guanidine scintigraphy; MRI, Magnetic resonance image; NET, Neuroendocrine tumor; SPECT, Single-photon emission computed tomography; SSTR, Somatostatin subtype receptor; STIR, Short TI Inversion Recovery; WBD, Whole-body diffusion; 5-HIAA, 5-hydroxyindoleacetic acid.

## Competing interests

The authors declare that they have no competing interests.

## Authors’ contributions

RJC: case preparation and revised the manuscript. The author read and approved the final manuscript; RB: case preparation, established the imaging protocols and revised the manuscript; BG: revised the manuscript; AT: revised the manuscript; FC: case preparation, drafted discussion and revised the manuscript. FC: is member of the advisor board member for the European Neuroendocrine Tumor Society. All the authors read and approved the final manuscript.
